# *Lactobacillus reuteri* NCHBL-005 improves wound healing by promoting the activation of fibroblasts through TLR2/MAPK signaling

**DOI:** 10.1186/s41232-025-00370-9

**Published:** 2025-04-10

**Authors:** Dong-Yeon Kim, Tae-Sung Lee, Yun-Ji Lee, So-Yeon Ahn, Byeongsam Chu, Do-Hyeon Jung, Yeong-Jun Kim, In-Su Seo, Wan-Gyu Kim, Young Jin Cho, Jung Joo Hong, Jong-Hwan Park

**Affiliations:** 1 National Primate Research Centre, Korea Research Institute of Bioscience and Biotechnology (KRIBB), Cheongju, Chungcheongbuk 28116 Republic of Korea; 2 Laboratory Animal Medicine, Animal Medical Institute, College of Veterinary Medicine, Chonnam National University, Buk-Gu, Gwangju 61186 Republic of Korea; 3 Nodcure, Inc., Buk-Gu, Gwangju 61186 Republic of Korea

**Keywords:** Wound healing, *L. reuteri* NCHBL-005, Fibroblast, MAPK, TLR2

## Abstract

**Background:**

Wound healing is a complex physiological process essential for restoring tissue integrity following various injuries, ranging from minor, everyday incidents to post-surgical complications. Emerging studies have demonstrated that lactic acid bacteria (LAB) can offer benefits beyond gut health, extending their positive effects on skin health. This study investigated the potential of *Lactobacillus reuteri* NCHBL-005, a honeybee-derived probiotic strain, to enhance fibroblast-mediated wound healing.

**Method:**

L929 cells and mouse embryonic fibroblasts (MEFs) were utilized as models to specifically target fibroblasts. To assess the wound healing potential in vitro, a scratch assay was performed, providing insights into wound closure. Additionally, we created wound models in mice to evaluate the in vivo effects of the treatment.

**Results:**

Our results showed that *L. reuteri* NCHBL-005 significantly accelerated wound closure in L929 fibroblast compared to other lactobacilli and exhibited superior efficacy in activating the mitogen-activated protein kinase (MAPK) pathway. Through MAPK inhibition assays, we confirmed that the wound healing effects of *L. reuteri* NCHBL-005 were MAPK-dependent, promoting fibroblast proliferation and differentiation. Notably, *L. reuteri* NCHBL-005 treatment did not facilitate wound healing in MEF cells derived from Toll-like-receptor 2 knockout (TLR2^−/−^) mice, highlighting the critical role of TLR2 in this mechanism. In vivo studies further corroborated these findings, in which topical administration of *L. reuteri* NCHBL-005 enhanced wound healing and stimulated fibroblast proliferation and activation, as confirmed by histopathological analysis.

**Conclusion:**

These findings revealed that *L. reuteri* NCHBL-005 activates fibroblasts through TLR2 stimulation and subsequent MAPK pathway activation, suggesting its potential as a promising therapeutic candidate for wound management.

**Supplementary Information:**

The online version contains supplementary material available at 10.1186/s41232-025-00370-9.

## Background

Fibroblasts, the most prevalent cell type in dermal connective tissues, play a pivotal role in maintaining the skin’s structural integrity and functional properties [1]. These cells are primarily responsible for producing collagen, extracellular matrix components, and proteins that provide skin with elasticity and strength [2]. The dynamic behavior of fibroblasts underscores their essential role in tissue homeostasis and wound healing, where they regulate regeneration by synthesizing signaling molecules—such as growth factors, cytokines, and chemokines—that interact with other cell types, including keratinocytes, endothelial cells, and immune cells, to coordinate the healing process [3].

During the proliferation phase of wound healing, activated fibroblasts migrate into the wound space, proliferate, and produce structure-related proteins while also secreting growth factors, cytokines, and signaling molecules essential for modulating the healing process [3]. Simultaneously, fibroblasts differentiate into myofibroblasts, a specialized cell type expressing alpha-smooth muscle actin (α-SMA), which generates contractile forces that promote wound closure by drawing the edges together [3, 4].

Given their central role, fibroblasts have been a focal point for therapeutic strategies aiming to enhance wound healing, particularly in difficult-to-treat clinical cases, such as chronic wounds, diabetic ulcers, and pressure sores with impaired healing ability [5]. Targeting fibroblasts through novel approaches, such as growth factor administration, cell-based therapy, or modulation of their signaling pathways, offers the potential to accelerate wound healing and improve clinical outcomes [3]. Thus, manipulating and supporting the functions of fibroblasts represents a critical therapeutic approach for the restoration and preservation of skin integrity and functionalities.

A substantial body of evidence indicates that LAB exerts beneficial effects on human health [6, 7]. In particular, growing research highlights their role in dermatological health and wound healing [8, 9]. Specifically, certain strains of LAB have shown significant efficacy in enhancing wound healing by upregulating collagen synthesis, promoting skin regeneration, facilitating wound closure, and modulating the inflammatory response [10]. For instance, *Lactobacillus rhamnosus* GG has been reported to facilitate re-epithelialization by increasing keratinocyte proliferation and promoting migration through the upregulation of CXCL2 and its receptor CXCR2, while *L. rhamnosus* LR has been found to strengthen skin barrier functions by restoring the human epidermis [11, 12]. *L. reuteri* ATCC-55730 exhibits anti-inflammatory effects by inhibiting the transcription factors for interleukin-8 and human beta-defensin-2 in infected epithelial cells [13]. Beyond their wound-healing properties, LAB strains have demonstrated potent anti-pathogenic effects. Studies have shown that *L. rhamnosus* GG can effectively suppress *S. aureus* infections in keratinocytes by hindering bacterial growth and adherence [14]. LAB strains, including *L. rhamnosus* and *L. plantarum*, have been also suggested to promote angiogenesis, a critical process in wound healing, by upregulating the expression of vascular endothelial growth factor and/or epidermal growth factor [15]. Collectively, these findings suggest that lactic acid bacteria could serve as suitable candidates for the development of wound healing therapies.

In this study, we investigated the wound-healing effects of *L. reuteri* NCHBL-005, isolated from the intestine of a honeybee in L929 fibroblasts and MEFs. Furthermore, we aimed to elucidate the wound-healing potential of *L. reuteri* NCHBL-005 in vivo by applying it topically to BALB/c mice with experimentally induced skin wounds.

## Methods

### Cell culture

L929 mouse fibroblast cells (ATCC CCL-1) were cultured in Iscove’s modified Dulbecco’s medium (IMDM; Thermo Fisher Scientific, Waltham, MA, USA), supplemented with 10% heat-inactivated fetal bovine serum (FBS) and 1% penicillin–streptomycin (P.S.; Thermo Fisher Scientific). Wild-type (WT) and TLR2^−/−^ C57BL/6 J mouse embryos were utilized to generate MEFs. On gestational days 13 or 14, embryos were isolated, tissues were minced, and cells were dissociated with trypsin–EDTA [16]. MEFs were cultured in Dulbecco’s modified Eagle’s medium (DMEM; Welgene, Gyeongsan, South Korea) supplemented with 10% FBS and 1% P.S.

### Isolation and identification of lactobacilli in honeybees

Twenty-five worker honeybees were collected from Gwangju, South Korea. The midguts of homogenized and plated on de Man, Rogosa, and Sharpe (MRS) agar (Oxoid, Hampshire, UK) for anaerobic incubation at 37 ℃ for 3–4 days. A total of 100 colonies were subcultured, followed by genomic DNA extraction using a DNA extraction kit (QIAGEN, Hilden, Germany). The 16S rDNA gene was then amplified and sequenced for species identification. The resulting sequences were compared with those in the NCBI BLAST database, leading to the identification of the following three *Lactobacillus* strains: *L. kunkeei* NCHBL-003 (KCTC 14908BP), *L. plantarum* NCHBL-004 (KCTC 14909BP), and *L. reuteri* NCHBL-005 (KCTC 15449BP). These *Lactobacilli* were boiled, lyophilized, and diluted in D-PBS for the experiment.

### Cell scratch assay

L929 and MEFs were seeded in 12-well plates (2 $\times$ 10^5^ cells per well) for a scratch wound healing assay. After 80% confluence, a uniform scratch was created across the cell monolayer using a sterile P1000 pipette tip to establish the wound. The cells were subsequently rinsed with PBS to remove any cellular debris generated from scratching. Fresh culture media—IMDM for L929 fibroblasts or DMEM for MEFs—supplemented with either 2% FBS or 10 μg/ml *Lactobacillus* spp. were then added to each well. Pam3CSK4 was treated at a concentration of 10 ng/ml. Scratch closure was monitored at 24, 48, and 72 h, and analyzed with ImageJ (version 1.8.0, National Institutes of Health, Bethesda, MD, USA).

### Methyl-thiazolyl-tetrazolium (MTT) cell proliferation assay

MTT (4 mg/ml; Sigma-Aldrich, St. Louis, MO, USA) was prepared in D-PBS and added to cells in IMDM with 10% MTT stock. After a 4-h incubation, the MTT-containing medium was gently removed, and dimethyl sulfoxide (DMSO; Sigma-Aldrich) was added to each well to dissolve the formazan crystals. The absorbance of the resulting purple solution was measured at 570 nm using a microplate reader (BioTek, Winooski, VT, USA).

### Western blot

Cells were lysed using a lysis buffer supplemented with protease (Roche, Mannheim, Germany) and phosphatase inhibitors (Sigma-Aldrich). Proteins were separated by SDS-PAGE and transferred to a nitrocellulose membrane, and incubated overnight with primary antibodies targeting phosphorylated forms of p38 MAPK (p38; Cell signaling Technology, Beverly, MA, USA), c-jun N-terminal kinase (JNK; Cell signaling Technology) and extracellular signal-regulated kinase (ERK; Cell signaling Technology), and β-actin (Santa Cruz Biotechnology, Dallas, TX, USA). After incubation with HRP-conjugated secondary antibodies (Thermo Fisher Scientific), protein bands were visualized using Clarity Western ECL Substrate (Bio-Rad, Hercules, CA, USA) and quantified with ImageJ.

### Inhibitor assay

L929 fibroblasts were pre-treated for 2 h with specific MAPK inhibitors. The cells were incubated with 10 μM of each of the following inhibitors: the JNK inhibitor (SP600125; Calbiochem, La Jolla, CA, USA), the p38 MAPK inhibitor (SB203580; Selleck Chemicals, Houston, TX, USA), and the ERK inhibitor (PD0325901; Selleck Chemicals). Following the pre-treatment of inhibitors, the cells were treated with *L. reuteri* NCHBL-005 to assess their effects on wound healing.

### Lactate dehydrogenase (LDH) cell viability assay

L929 cells were seeded in 12-well plates at a density of 2 $\times$ 10^5^ cells/ml and exposed to *L. reuteri* NCHBL-005 and MAPK inhibitors for 24, 48, and 72 h. LDH levels were measured using CytoTox 96® Non-Radioactive Cytotoxicity Assay kits (Promega, Madison, WI, USA) according to the manufacturer’s instructions. For the lysis control, the lysis solution provided in the kit was applied.

### Immunofluorescence

L929 fibroblasts (2 $\times$ 10^5^ cells/well) were treated with 10 μg/ml of *Lactobacillus* spp. to assess protein expression. The cells were fixed with 4% formaldehyde and subsequently permeabilized with 0.1% Triton X-100 (Sigma-Aldrich). Following permeabilization, the cells were incubated with an anti-α-SMA antibody (Invitrogen, Carlsbad, CA, USA) for 2 h. The cell nuclei were counterstained with 4′,6-diamidino-2-phenylindole (DAPI; Invitrogen). Fluorescent images were obtained using a fluorescence microscope.

### HEK-Blue assay

HEK-Blue-hTLR2 cells (5 $\times$ 10^4^ per well) were plated in 96-well plates and incubated overnight. The next day, Lactobacillus strains were introduced, and the cells were maintained for 6 h. SEAP levels were assessed using the HEK-Blue™ Detection (Invivogen, San Diego, CA, USA), and the optical density was measured at 620–650 nm with a microplate reader.

### Animal experiment

Seven-week-old female BALB/c mice were purchased from Damul Science (Daejeon, South Korea). After acclimation, seven mice were designated for wound healing evaluation, and 30 for histological analysis (5 mice per day for each time point). Two full-thickness wounds were created on dorsal skin using a 4-mm biopsy punch (Kai Industries Co., Ltd., Gifu, Japan). To prevent wound contraction, silicon splints were affixed around each wound with cyanoacrylate adhesive and secured using interrupted 6–0 nylon sutures. A transparent, occlusive dressing film (OPSITE FLEXIFIX; Smith & Nephew, London, UK) was applied over each wound site to maintain a controlled environment. Wounds were photographed on days 0, 2, 4, 6, 8, and 10 post-wounding, coinciding with dressing change days. For evaluation, one wound was treated with D-PBS (Welgene), while the contralateral wound was treated with *L. reuteri* NCHBL-005 (10 μg/ml, 10 μl) according to the experimental schedule. Wound size was quantified from the photographic images using ImageJ.

### Histological analysis

Skin samples were collected on days 0, 2, 4, 6, 8, and 10 post-wounding, fixed in formalin, and paraffin-embedded. Sections were stained with hematoxylin and eosin for histological evaluation and Masson’s trichrome stain for collagen deposition. Epithelial normalization, cellular infiltration, and granulation were scored from 0 (absent) to 4 (marked). The scoring criteria for histologic evaluation are listed in Table 1. For immunohistochemistry, sections were stained with anti-Ki67 antibody (1:200 dilution; Cell signaling Technology) and detected using 3,3′-diaminobenzidine tetrahydrochloride hydrate (Tokyo Chemical Industry, Tokyo, Japan).

**Table 1:** Scoring index for histological evaluation

Score	Epithelial normalization	Granulation	Cell infiltration
0	No epithelialization	No granulation tissue	No infiltration
1	Thin, immature epithelium	Minimal formation	Minimal infiltration
2	Hyperplastic epithelium	Mild formation	Mild infiltration
3	Partial normalization	Moderate formation	Moderate infiltration
4	Complete normalization	Excessive formation	Severe infiltration

### Statistical analysis

GraphPad Prism (version 10.2, San Diego, CA, USA) was used for statistical analyses. Significant differences between the groups were determined using a two-way analysis of variance (ANOVA) and the Mann–Whitney *U* test.

## Results

### *L. reuteri* NCHBL-005 enhances wound healing through L929 proliferation and differentiation

**Fig. 1:**
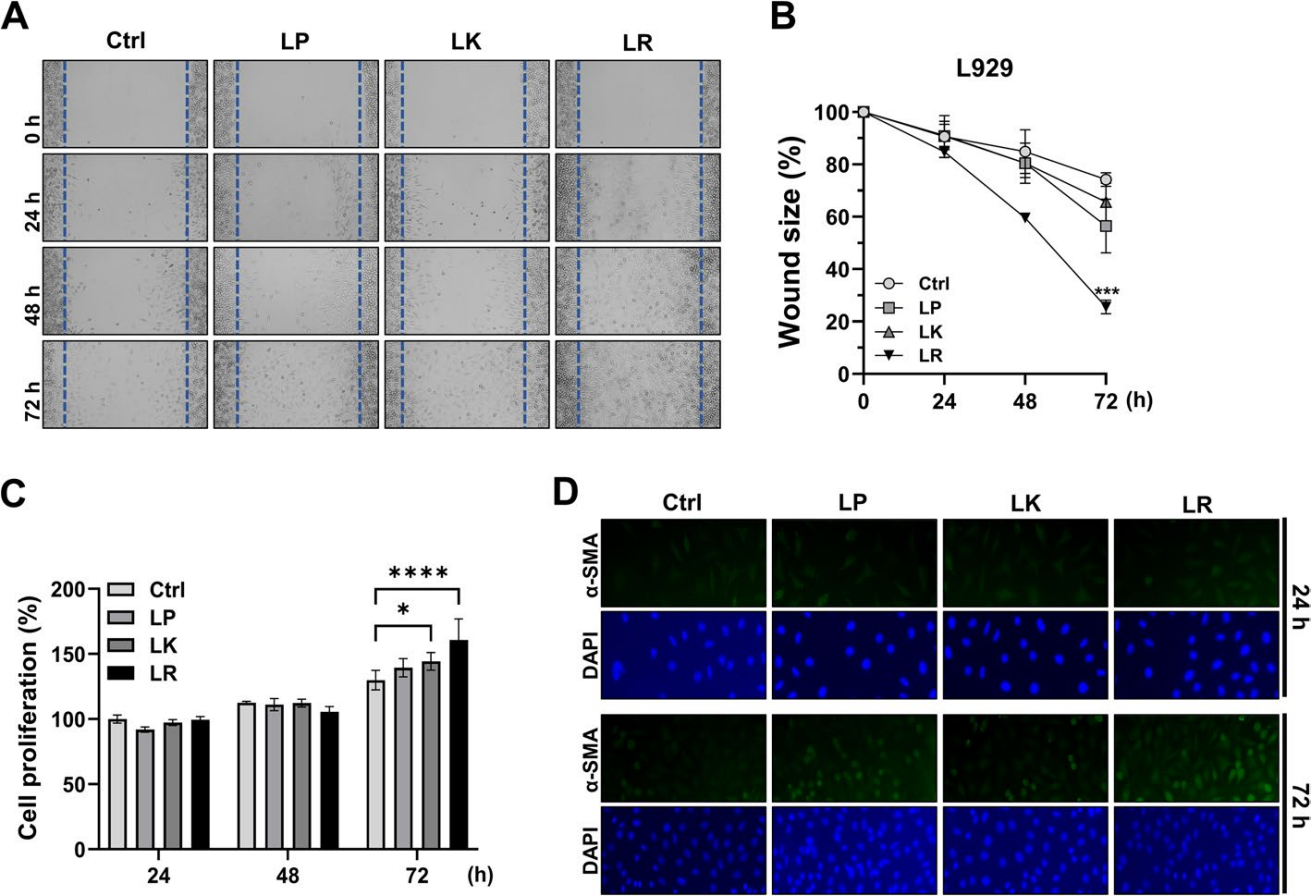
The wound healing effects of honeybee-derived *Lactobacilli* in fibroblasts. **A** Representative light microscope images from in vitro scratch assays conducted on L929 fibroblasts (0, 24, 48, and 72 h post-treatment). **B** Quantification of the wound area using Image J, with the scratch area at 0 h (no treatment) set as 100% (mean ± SD). **C** Proliferation of L929 fibroblasts treated with different strains of *lactobacilli* at 24, 48, and 72 h post-treatment (mean ± SD). **D** Immunofluorescence staining of α-SMA (green) and DAPI (blue) in L929 fibroblasts at 24 and 72 h post-treatment. The significance of differences among the groups was assessed using two-way ANOVA, with the level of significance set at *p < 0.05, ***p < 0.001, and ****p < 0.0001. Ctrl. Control; LP, *L. plantarum* NCHBL-004; LK, *L. kunkeei* NCHBL-003; LR, *L. reuteri* NCHBL-005; α-SMA, α-smooth muscle actin; DAPI, 4′,6-diamidino-2-phenylindole; ANOVA, analysis of variance; SD, standard deviation

A scratch assay was conducted using L929 fibroblasts to assess the wound-healing potential of three *Lactobacilli* strains isolated from honeybees: *L. reuteri* NCHBL-005, *L. kunkeei* NCHBL-003, and *L. plantarum* NCHBL-004. At 24 h, no significant differences were observed between the treated and control groups (Fig. 1A, B). However, by 48 h, treatment with *L. reuteri* NCHBL-005 led to a reduction in wound size, approaching statistical significance (*p* = 0.06) (Fig. 1A, B). At 72 h, this strain demonstrated a significant increase in fibroblast proliferation and migration, resulting in a pronounced reduction in wound size (Fig. 1A, B). While the other strains showed some wound closure effects, the differences were not statistically significant.

Fibroblast proliferation and differentiation into myofibroblasts are fundamental processes in wound healing [17, 18]. To further investigate, MTT, immunofluorescence, and western blot analyses were conducted. While no significant differences in cell proliferation were observed at 24 and 48 h, *L. reuteri* NCHBL-005 significantly enhanced proliferation at 72 h (Fig. 1C). *L. kunkeei* NCHBL-003 also increased cell proliferation but to a lesser extent (Fig. 1C). Moreover, *L. reuteri* NCHBL-005 robustly promoted fibroblast differentiation into myofibroblasts, as evidenced by elevated α-SMA expression at 72 h, confirmed by immunofluorescence (Fig. 1D). These findings suggest that *L. reuteri* NCHBL-005 has superior potential to enhance both fibroblast proliferation and differentiation.

### MAPK signaling is essential for *L. reuteri* NCHBL-005-mediated wound healing in L929

**Fig. 2:**
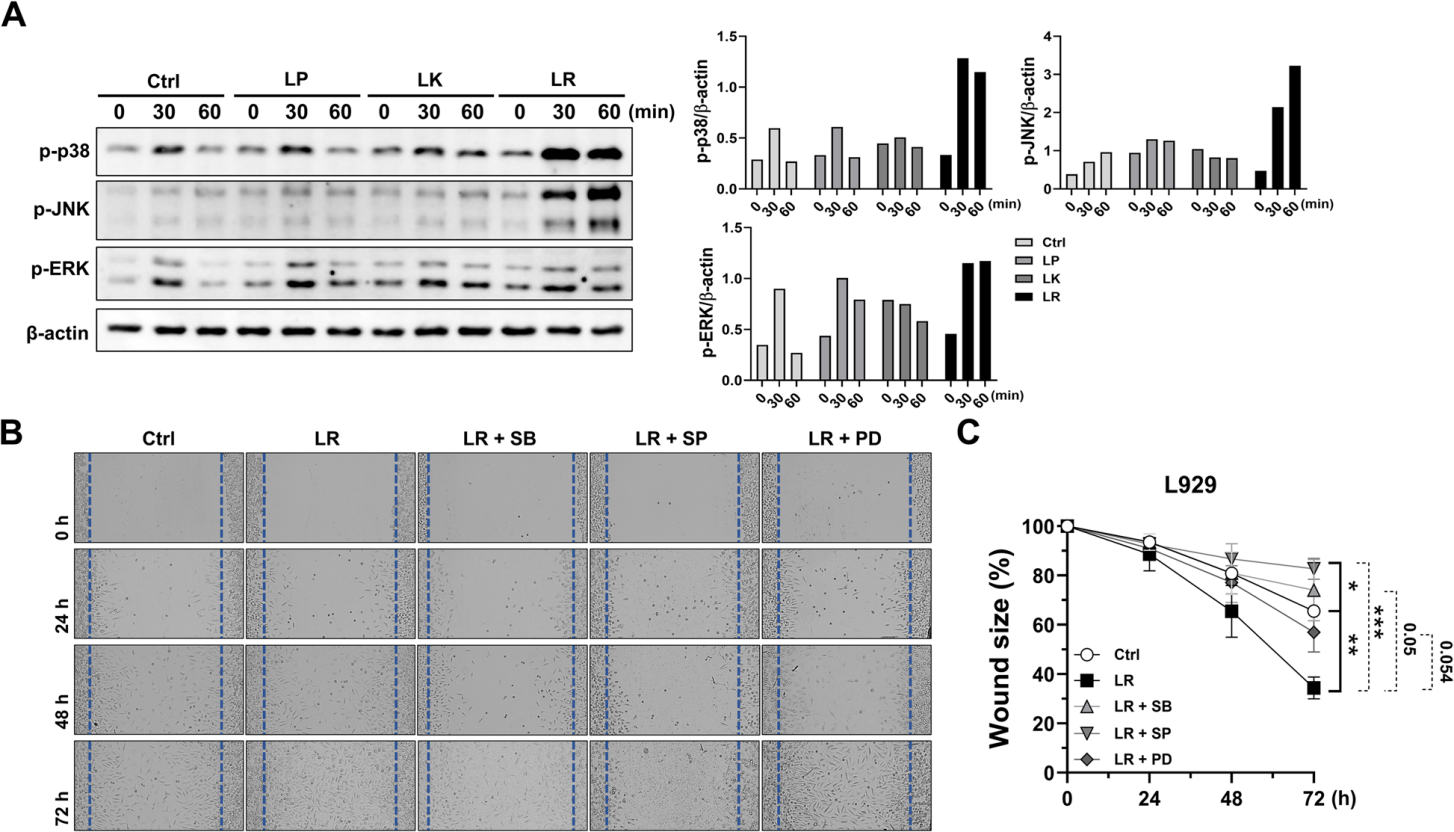
The modulation of MAPK phosphorylation and wound healing by *L. reuteri* NCHBL-005. **A** Expression of phosphorylated p38, ERK, and JNK detected by western blot analysis. **B** Representative light microscope images from in vitro scratch assays conducted on L929 fibroblasts treated with SB203580, SP600125, and PD0325901 at 24, 48, and 72 h post-treatment. **C** Quantification of the wound area using Image J, with the scratch area at 0 h (no treatment) set as 100% (mean ± SD). The significance of differences among the groups was assessed using two-way ANOVA, with the level of significance set at *p < 0.05, **p < 0.01, and ***p < 0.001. Ctrl. Control; LP, *L. plantarum* NCHBL-004; LK, *L. kunkeei* NCHBL-003; LR, *L. reuteri* NCHBL-005; p-p38, phosphorylated p38 mitogen-activated protein kinase; p-JNK, phosphorylated c-jun N-terminal kinase; p-ERK, phosphorylated extracellular signal-regulated kinase; β-actin, beta-actin; SB, SB203580; SP, SP600125; PD, PD0325901; ANOVA, analysis of variance; SD, standard deviation

MAPK is a key regulator of intracellular signaling controlling cellular growth, proliferation, and differentiation [19]. In our study, we focused on the phosphorylation of MAPK to explore the mechanisms behind the activation of L929 fibroblasts induced by lactobacilli derived from honeybees. Our results showed that treatment with *L. reuteri* NCHBL-005 led to a greater enhancement of p38 and JNK phosphorylation compared to the other strains (Fig. 2A). Although the other strains also induced phosphorylation of these proteins, they were less effective than *L. reuteri* NCHBL-005 (Fig. 2A). In contrast, ERK phosphorylation exhibited a relatively modest increase (Fig. 2A). These results were consistently observed in repeated experiment (Fig. S1). To determine the role of MAPK signaling in the wound healing effects of *L. reuteri* NCHBL-005, specific inhibitors were utilized: SB203580, SP600125, and PD0325901. The results indicated that all three inhibitors significantly reduced the wound-healing effects of *L. reuteri* NCHBL-005, confirming the involvement of MAPK signaling in this process (Fig. 2B, C). The SP600125 showed the most substantial inhibition of wound healing promoted by *L. reuteri* NCHBL-005 (Fig. 2B, C).

### *L. reuteri* NCHBL-005 promotes the proliferation and differentiation of L929 through MAPK signaling

**Fig. 3:**
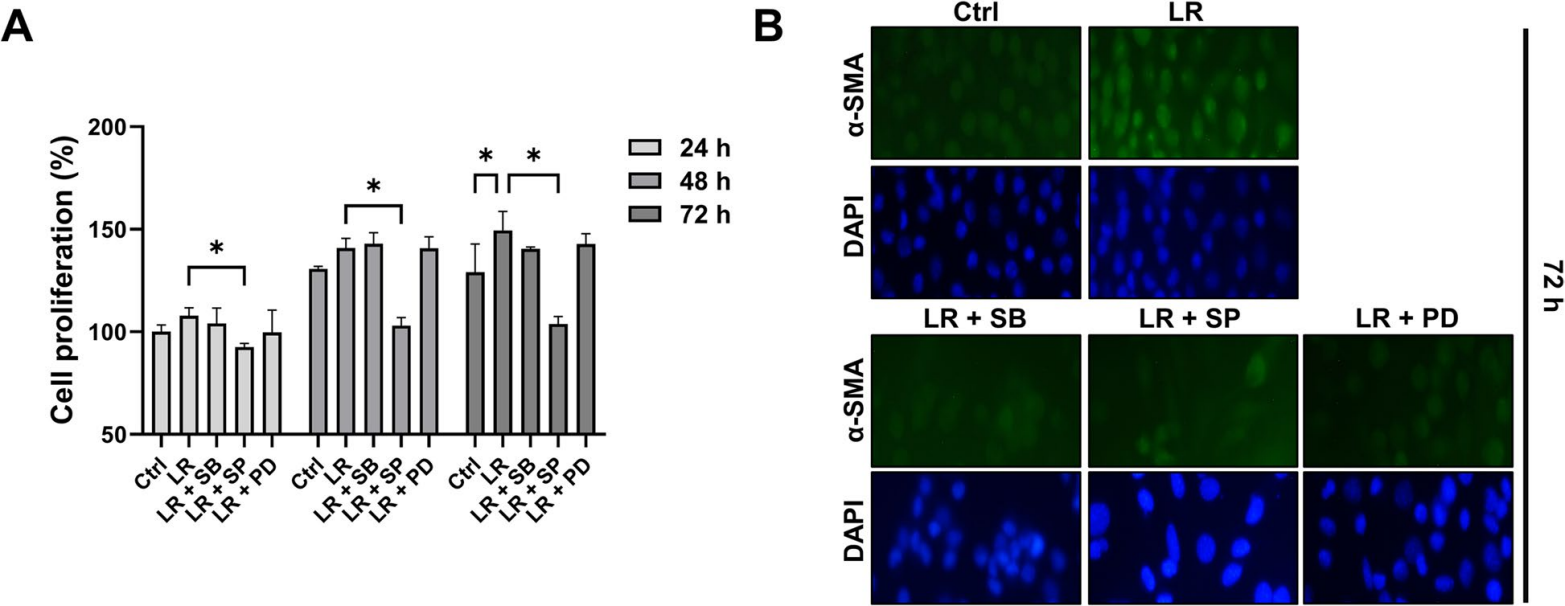
Suppression of *L. reuteri* NCHBL-005-induced fibroblast proliferation and differentiation through MAPK inhibition. **A** Proliferation of L929 fibroblasts treated with MAPK inhibitors and *L. reuteri* NCHBL-005 at 24, 48, and 72 h post-treatment (mean ± SD). **B** Immunofluorescence staining of α-SMA (green) and DAPI (blue) in L929 fibroblasts. The significance of differences among the groups was assessed using two-way ANOVA, with the level of significance set at *p < 0.05. Ctrl. Control; LR, *L. reuteri* NCHBL-005; SB, SB203580; SP, SP600125; PD, PD0325901; α-SMA, α-smooth muscle actin; DAPI, 4′,6-diamidino-2-phenylindole; ANOVA, analysis of variance; SD, standard deviation

We further investigated whether MAPKs signaling contributes to the proliferation of L929 fibroblasts and their differentiation into myofibroblasts. The MTT assay showed a significant decrease in proliferation at each time point following treatment with SP600125 compared to *L. reuteri* NCHBL-005 treated group, whereas SB203580 and PD0325901 had no such effect (Fig. 3A). Cytotoxicity effects were not detected under any of the experimental conditions tested (Fig. S2). Immunofluorescence results revealed that *L. reuteri* NCHBL-005-induced α-SMA expression was significantly reduced by all inhibitors (Fig. 3B). These findings indicate that JNK plays a specific role in cell proliferation, whereas all three MAPK pathways are involved in the regulation of differentiation.

### TLR2 is required for *L. reuteri* NCHBL-005-mediated wound healing in fibroblast

**Fig. 4:**
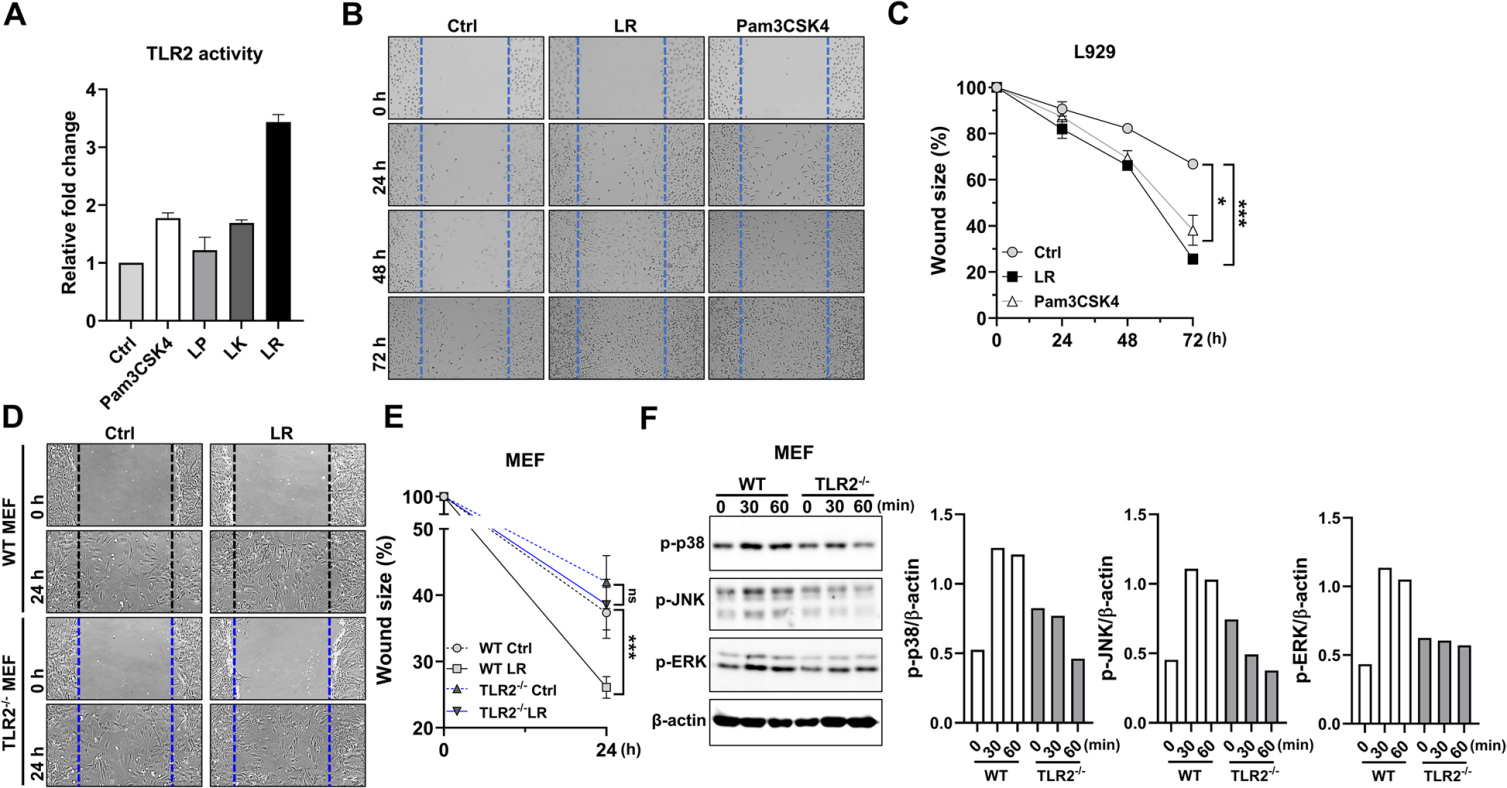
TLR2-dependent wound healing and MAPK phosphorylation by *L. reuteri* NCHBL-005. **A** TLR2 activity of *Lactobacilli*. **B** Representative light microscope images from in vitro scratch assays conducted on L929 fibroblasts (0, 24, 48, and 72 h post-treatment). **C** Quantification of the wound area using Image J, with the scratch area at 0 h (no treatment) set as 100% (mean ± SD). **D** Representative light microscope images from in vitro scratch assays conducted on MEFs at 0 and 24 h post-treatment. **E** Quantification of the wound area using Image J, with the scratch area at 0 h (no treatment) set as 100% (mean ± SD). **F** Expression of phosphorylated p38, ERK, and JNK detected by western blot analysis. The significance of differences among the groups was assessed using two-way ANOVA, with the level of significance set at *p < 0.05 and ***p < 0.001. Ctrl, control; MEF, mouse embryonic fibroblast; WT, wild-type; TLR2, toll-like receptor 2; LR, *L. reuteri* NCHBL-005; p-p38, phosphorylated p38 mitogen-activated protein kinase; p-JNK, phosphorylated c-jun N-terminal kinase; p-ERK, phosphorylated extracellular signal-regulated kinase; β-actin, beta-actin; ANOVA, analysis of variance; SD, standard deviation

The cell wall structure of lactobacilli, such as *L. reuteri* NCHBL-005, engages innate immune receptors, including TLR2, initiating upstream signaling in the MAPK pathway and driving diverse cellular activation processes [20]. Previous studies have suggested that TLR2 plays a critical role in fibroblast activation and tissue regeneration [21]. In light of this evidence, we hypothesized that TLR2 might be a key mediator in the wound-healing effects of *L. reuteri* NCHBL-005. The ability of three *Lactobacillus* strains to activate TLR2 was evaluated, with *L. reuteri* NCHBL-05 showing the strongest activation among the strains (Fig. 4A). To further investigate the role of TLR2 in wound healing, we assessed the effects of Pam3CSK4, a TLR2 ligand, on wound healing in L929 cells. Treatment with Pam3CSK4 significantly enhanced wound closure, producing effects comparable to those of *L. reuteri* NCHBL-005 (Fig. 4B, C). Additionally, the TLR2-dependent wound-healing effects of *L. reuteri* NCHBL-005 were analyzed using MEFs derived from WT and TLR2^−/−^ mice. WT MEFs treated with *L. reuteri* NCHBL-005 exhibited significantly enhanced wound closure compared to untreated controls, with noticeable wound size reduction at 24 h (Fig. 4D, E). Conversely, TLR2^−/−^ MEFs showed no significant improvement in wound healing (Fig. 4D, E). MAPK signaling analysis demonstrated increased phosphorylation of p38, JNK, and ERK in WT MEFs following *L. reuteri* NCHBL-005 treatment, whereas TLR2^-/-^ MEFs showed no MAPK activation (Fig. 4F). In untreated MEFs, there was no noticeable difference in phosphorylation levels between WT and TLR2^−/−^ MEFs (Fig. S3). These results suggest that the wound-healing effects of *L. reuteri* NCHBL-005 are mediated by TLR2-dependent activation of the MAPK pathway.

### *L. reuteri NCHBL-005 *accelerates in vivo wound healing in a murine wound model

**Fig. 5:**
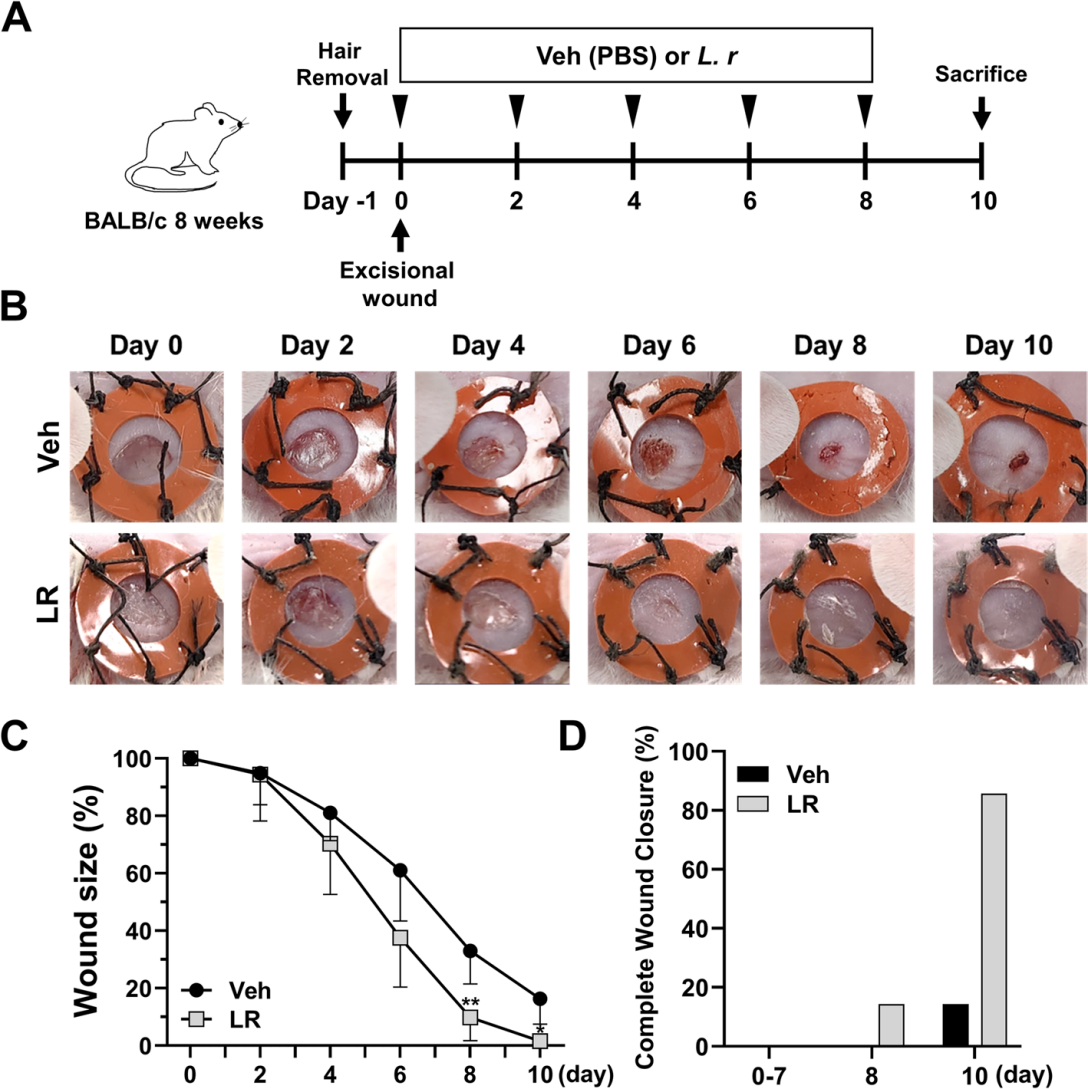
Effects of *L. reuteri* NCHBL-005 on wound healing in mouse model. **A** Schematic timeline of the experiment. **B** Representative images of the wound sites treated with vehicle or *L. reuteri* NCHBL-005. **C** Quantitative analysis of wound size reduction (mean ± SD). **D** Percent of mouse subjects with completely closed wounds. The significance of differences among the groups was assessed using two-way ANOVA, with the level of significance set at *p < 0.05 and **p < 0.01. Veh, Vehicle; LR, *L. reuteri* NCHBL-005; ANOVA, analysis of variance; SD, standard deviation

To assess the in vivo wound healing effects of *L. reuteri* NCHBL-005, two excisional wounds were created on the dorsal skin of mice, with topical treatment as outlined in Fig. 5A. The left wound was treated with D-PBS (vehicle), while the right wound was treated with *L. reuteri* NCHBL-005. No significant difference in wound size was observed between the two groups for up to day 2 post-treatment. However, starting on day 4 post-treatment, the wounds treated with *L. reuteri* NCHBL-005 consistently exhibited a smaller wound size compared to the vehicle group (Fig. 5B, C). On days 8 and 10, significant reductions in wound size were observed for the *L. reuteri* NCHBL-005-treated wounds compared to the vehicle group (Fig. 5B, C). Complete wound closure was achieved in one subject by day 8 and in the other six subjects by day 10 in the *L. reuteri* NCHBL-005 group, whereas only one subject in the vehicle group achieved complete closure by day 10 (Fig. 5D). These findings demonstrate that topical treatment of *L. reuteri* NCHBL-005 could enhance in vivo wound healing.

### Treatment of *L. reuteri* NCBHL-005 promotes tissue regeneration during wound healing

**Fig. 6:**
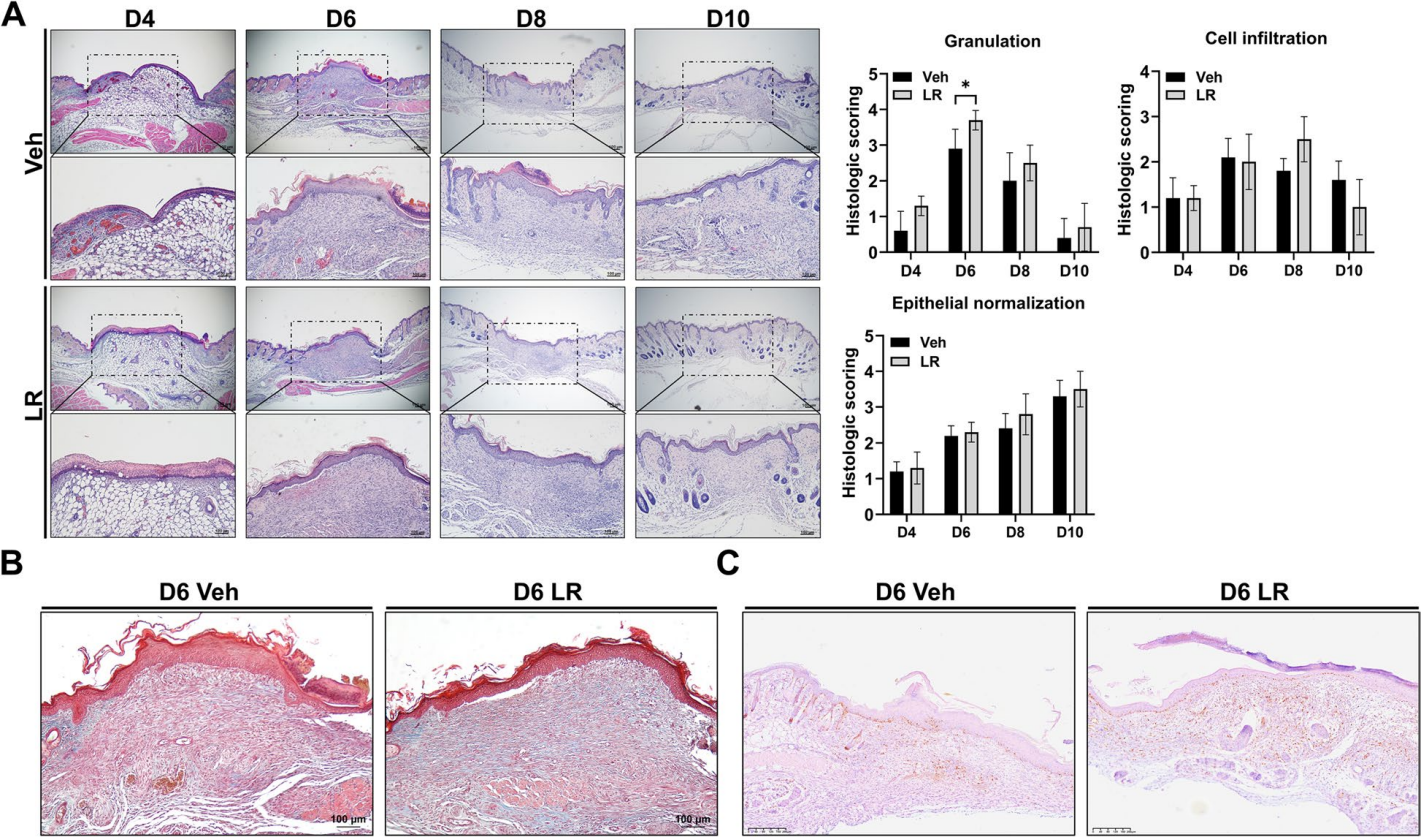
Histologic evaluation of mouse wounds treated with *L. reuteri* NCHBL-005. **A** Representative images of Hematoxylin and eosin-stained wounds treated with either vehicle or *L. reuteri* NCHBL-005 and evaluation of histologic evaluation of mouse wounds on days 4, 6, 8, and 10. **B** Representative images of Masson’s trichrome-stained wounds treated with either vehicle or *L. reuteri* NCHBL-005 on day 6 (mean ± SD). **C** Representative images of Ki-67 immuno-stained wounds treated with either vehicle or *L. reuteri* NCHBL-005 on day 6. The significance of differences among the groups was assessed using the Mann–Whitney *U* test, with the level of significance set at *p < 0.05. Veh, Vehicle; LR, *L. reuteri* NCHBL-005

Histological analysis of mouse skin wounds treated with *L. reuteri* NCHBL-005 was performed on days 0, 2, 4, 6, 8, and 10 to assess the effects on wound healing and tissue regeneration. Early re-epithelialization was observed by day 2 in both the vehicle and *L. reuteri* NCHBL-005 groups (data not shown). By day 4, re-epithelialization was nearly complete, but the *L. reuteri* NCHBL-005 group showed thicker clots, indicating enhanced repair (Fig. 6A). By day 6, granulation tissue was more organized in the *L. reuteri* NCHBL-005 group, with significantly higher collagen deposition, as shown by Masson’s trichrome staining, suggesting increased fibroblast activity (Fig. 6A, B). By day 8, the *L. reuteri* NCHBL-005 group exhibited faster epithelial layer normalization and increased inflammatory cell infiltration. By day 10, hair follicle formation and a decrease in inflammatory cells were noted in the *L. reuteri* NCHBL-005 group, indicating advanced wound healing (Fig. 6A).

Immunohistochemical analysis revealed a higher density of Ki-67, a cell proliferation marker, in the dermal zone of the *L. reuteri* NCHBL-005-treated group compared to the vehicle group, indicating an enhanced proliferative response (Fig. 6C). The results showed that *L. reuteri* NCHBL-005 significantly enhances wound healing by modulating granulation tissue formation, collagen deposition, and the inflammatory response in vivo.

## Discussion

Numerous studies on LAB have suggested their utility for various biomedical applications [7, 22, 23]. In this study, we elucidated the detailed mechanisms underlying the wound-healing effects of *L. reuteri* NCHBL-005. The results demonstrated that among the strains tested, *L. reuteri* NCHBL-005 most effectively enhances fibroblast activation needed for differentiation and proliferation. This enhancement was attributed to the activation of the MAPK signaling pathway. Specifically, it was demonstrated that *L. reuteri* NCHBL-005 activates the MAPK proteins in a TLR2-dependent manner in MEFs. Furthermore, the significant wound-healing effects of *L. reuteri* NCHBL-005 were also demonstrated in vivo.

Wound healing is a complex, multi-phase physiological process that involves hemostasis, inflammation, proliferation, and remodeling [24]. The proliferation phase is crucial for wound healing, as fibroblast activation drives key processes such as angiogenesis, extracellular matrix formation, re-epithelialization, and wound contraction [25]. Our study focused on elucidating the fibroblast-activating effects of *L. reuteri* NCHBL-005. We found that *L. reuteri* NCHBL-005 promotes fibroblast proliferation and differentiation into myofibroblasts, evidenced by increased cell proliferation and α-SMA expression, thereby supporting wound healing. Chronic ulcers and fibrosis, both fibroblast-associated wound pathologies, present substantial clinical challenges, underscoring the urgent need for more effective therapeutic strategies. Recent studies have demonstrated that fibroblasts comprise various subpopulations, each with distinct morphological and functional characteristics that uniquely influence wound healing [26, 27]. Understanding these fibroblast subtypes requires a comprehensive characterization of specific surface markers and various functional assays. The insights into these subtypes may contribute to the development of new therapeutics, including *L. reuteri* NCHBL-005, that can modulate the activity of fibroblasts to enhance wound healing and tissue regeneration while minimizing fibrosis and scarring.

In this study, we investigated the role of the MAPK signaling pathway in fibroblasts using L929 and MEFs. Our results showed that *L. reuteri* NCHBL-005 significantly activates the MAPK pathway, promoting their proliferation and differentiation, as confirmed by MAPK inhibitors. The MAPK signaling plays a crucial role in regulating various physiological processes, such as growth, differentiation, inflammation, and apoptosis, by transmitting signals from the cell surface to the nucleus in response to stimuli [19]. Dysregulated MAPK signaling is closely related to abnormal wound healing, which can manifest in two forms: excessive healing (e.g., keloid and hypertrophic scarring) and deficient healing (e.g., diabetic foot ulcers and venous leg ulcers). Excessive MAPK activation has been shown to contribute to conditions such as keloid formation [28–30], while reduced p38/MAPK activity is associated with impaired healing seen in conditions like diabetic foot ulcers [31]. Additionally, the differentiation of fibroblasts into myofibroblasts has been shown to depend on MAPK signaling [32, 33]. Moreover, a study has demonstrated that wound healing is delayed in MAPK-deficient models, further underscoring the essential role of this pathway in effective tissue repair [34]. Given these findings, further research is necessary to focus on optimizing the dosage of *L. reuteri* NCHBL-005 to effectively activate the MAPK pathway and achieve ideal wound healing outcomes.

We demonstrated that TLR2 can be activated by *L. reuteri* NCHBL-005, leading to the initiation of the MAPK pathway, fibroblast activation, and promotion of wound healing. Previous research suggests that TLR2 deficiency accelerates wound closure in diabetic mice, while similar studies have shown that inhibiting TLR2 can reduce inflammatory responses and mitigate renal ischemic injury following ischemia/reperfusion [35, 36]. Conversely, knocking out TLR2 has been reported to delay wound healing, highlighting the context-dependent role of TLR2 in specific conditions [37]. For instance, the deletion of TLR2 in endothelial cells has been shown to impair angiogenesis, resulting in delayed wound healing [38]. Additionally, Deiters et al. found that topical application of a TLR2 agonist, macrophage-activating lipopeptide-2, accelerated wound healing in diabetic murine skin wounds [39]. Although the distinct roles of TLR2 in wound healing are still under debate, current evidence indicates that its effects may differ significantly depending on the specific cellular targets. Our study contributes to this body of knowledge by demonstrating that *L. reuteri* NCHBL-005 activates the MAPK pathway via TLR2, thus promoting fibroblast activation and wound healing. Meanwhile, *L. reuteri* NCHBL-005 contains various ligands capable of stimulating not only TLR2 but also other innate immune receptors, potentially contributing to the promotion of wound healing through their activation. Among these, Nod2, a key receptor recognizing muramyl dipeptide—a conserved component of peptidoglycan—is likely to be engaged by *Lactobacillus*, a Gram-positive bacterium with a thick peptidoglycan layer abundant in such ligands [40]. Although direct evidence linking *L. reuteri* to Nod2 activation in the context of wound healing is limited, previous studies have shown that *L. reuteri* possesses strong Nod2 activity [41], suggesting a potential role for Nod2 in mediating its effects. In conclusion, our findings suggest that the TLR2/MAPK axis in fibroblasts may serve as a potential therapeutic target for enhancing wound healing. Furthermore, the potential involvement of other innate immune receptors, such as Nod2, warrants further investigation to elucidate the complex mechanisms underlying the multifaceted effects of *L. reuteri* NCHBL-005 in wound healing.

In murine models, wound healing is predominantly characterized by wound contraction, whereas in humans, the process is primarily driven by re-epithelialization and granulation tissue formation [42]. In this study, full-thickness circular wounds extending to the panniculus carnosus were surgically induced in mice. To prevent wound contraction and better replicate the human wound healing process, silicone splints were sutured to the wound edges. Consequently, wound repair in this model was mediated by mechanisms analogous to those observed in human tissue repair, particularly involving the formation of granulation tissue and re-epithelialization at the wound margins. Our study specifically focused on the activation of fibroblasts, as their role was deemed crucial in the formation of granulation tissue, which is essential for effective wound healing. Given that rapid granulation tissue formation can significantly accelerate the wound healing process, this model was particularly well-suited for our investigation. The results indeed demonstrated that *L. reuteri* NCHBL-005 can significantly enhance granulation tissue formation, thereby accelerating the wound healing process.

This study has several limitations. First, it focused solely on the healing of surgically induced wounds, making it difficult to extrapolate the result to other wound types, such as diabetic wounds or those delayed by bacterial infections. Second, the therapeutic effects of *L. reuteri* NCHBL-005 are likely due to the intricate microbe-host interactions, as the strain produces a diverse array of bioactive molecules. Therefore, to reduce the risk of potential side effects and enhance therapeutic specificity, it may be necessary to isolate and identify specific components of *L. reuteri* NCHBL-005.

In conclusion, this study demonstrates the wound-healing effects of *L. reuteri* NCHBL-005 via the TLR2/MAPK signaling pathway. The bacterium’s therapeutic potential was validated in an in vivo model, highlighting the importance of targeting fibroblast TLR2/MAPK pathways in the development of advanced therapeutic strategies for tissue repair.

## Supplementary Information

Supplementary Material 1: Figure S1. Repetition experiment of Figure 2A

Supplementary Material 2: Figure S2. Evaluation of LDH Release with MAPK Inhibitor and Lactobacillus reuteri NCHBL-005

Supplementary Material 3: Figure S3. Expression of phosphorylated p38, ERK, and JNK on untreated WT and TLR2-/- MEFs

## Data Availability

The datasets used and/or analyzed during the current study are available from the corresponding author on reasonable request.

## Abbreviations

ERK: Extracellular signal-regulated kinase
JNK: c-jun N-terminal kinase
MAPK: Mitogen-activated protein kinase
MEF: Mouse embryonic fibroblast
p38: P38 mitogen-activated protein kinase
α-SMA: Alpha-smooth muscle actin
TLR2: Toll-like receptor 2
