# Supplementary material for: Lactobacillus reuteri NCHBL-005 improves wound healing by promoting the activation of fibroblasts through TLR2/MAPK signaling
Source: Inflamm Regen. 2025 Apr 10;45:10. doi: 10.1186/s41232-025-00370-9 (PMC11983859; doi:10.1186/s41232-025-00370-9)
Supplement: Supplementary file 2 — Supplementary Material 2: Figure S2. Evaluation of LDH Release with MAPK Inhibitor and Lactobacillus reuteri NCHBL-005 [file 41232_2025_370_MOESM2_ESM.docx]

**Supplementary data**


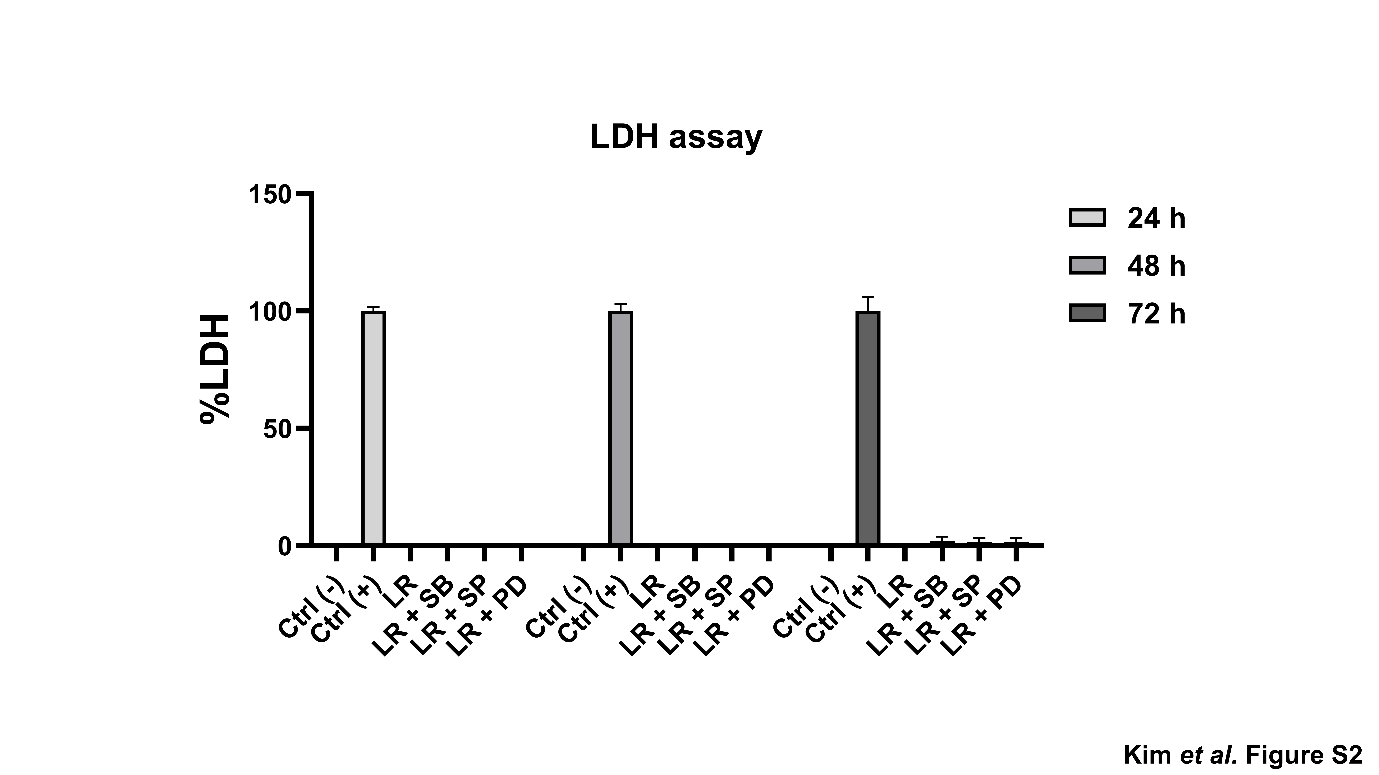


**Supplementary figure 2. Evaluation of LDH Release with MAPK Inhibitor and *Lactobacillus reuteri* NCHBL-005**

Cell viability was assessed using the LDH release assay on supernatants collected at 24, 48, and 72 hours from the following treatment groups: Control, LR, LR + SB, LR + SP, and LR + PD. LDH. Lactate dehydrogenase; Ctrl. Control; LR, *L. reuteri* NCHBL-005; SB, SB203580; SP, SP600125; PD, PD0325901
