# Supplementary material for: Lactobacillus reuteri NCHBL-005 improves wound healing by promoting the activation of fibroblasts through TLR2/MAPK signaling
Source: Inflamm Regen. 2025 Apr 10;45:10. doi: 10.1186/s41232-025-00370-9 (PMC11983859; doi:10.1186/s41232-025-00370-9)
Supplement: Supplementary file 3 — Supplementary Material 3: Figure S3. Expression of phosphorylated p38, ERK, and JNK on untreated WT and TLR2-/- MEFs [file 41232_2025_370_MOESM3_ESM.docx]

**Supplementary data**


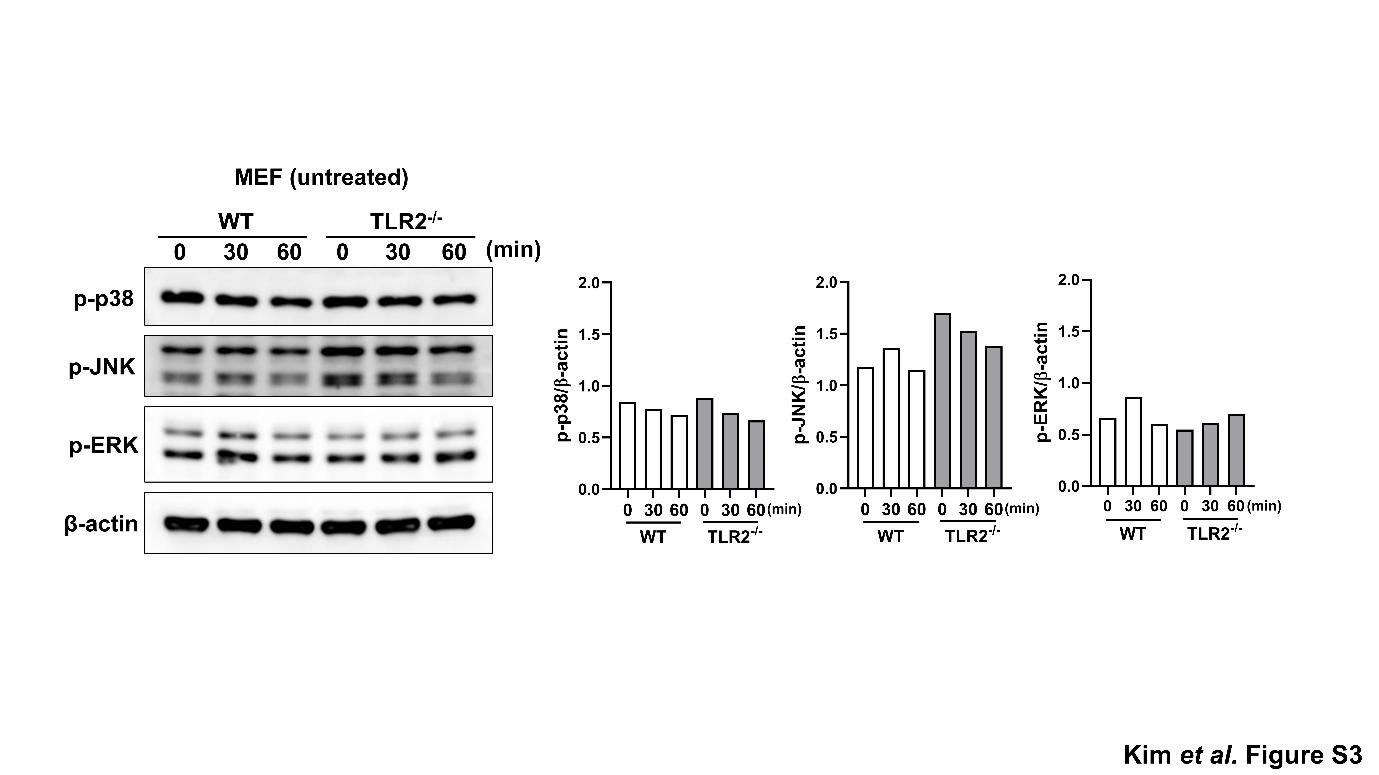


**Supplementary figure 3. Expression of phosphorylated p38, ERK, and JNK on untreated WT and TLR2^-/-^ MEFs.**

Expression of phosphorylated p38, ERK, and JNK detected by western blot analysis.
